# Retention of iron and zinc in yam flour and boiled yam processed from white yam (*D. rotundata*) varieties

**DOI:** 10.1002/fsn3.445

**Published:** 2016-12-05

**Authors:** Busie Maziya‐Dixon, Emmanuel Oladeji Alamu, Faustina Dufie Wireko‐Manu, Asiedu Robert

**Affiliations:** ^1^ Food and Nutrition Sciences LaboratoryInternational Institute of Tropical AgricultureIbadanOyo StateNigeria; ^2^ Food and Nutrition Sciences LaboratoryInternational Institute of Tropical Agriculture Southern Africa Research and Administration Hub (SARAH) CampusChelstonLusakaZambia; ^3^ Department of Food Science and TechnologyKwame Nkrumah University of Science and TechnologyKumasiGhana

**Keywords:** *D. rotundata*, micronutrient, physicochemical, processing, staple crops

## Abstract

This study investigated the impact of processing on retention of iron and zinc in *D. rotundata*. Fresh tubers were processed into boiled yam and yam flour and analyzed for zinc, iron, and physicochemical properties. Percent true retention (%TR) was assessed using paired samples and a formula that compensated for loss or gain of moisture and soluble solids. The retention of iron ranged from 55.5% to 98.7% in boiled yam and 25.2% to 54.9% in yam flour; retention of zinc ranged from 49.3% to 97.5% in boiled yam and 18. 9% to 43.1% in yam flour. The amount of iron retained in boiled yam correlated with the amount in the fresh samples (*r* = .79), likewise in yam flour (*r* = .82). A similar trend was observed for zinc. From our study, we conclude that retention of iron and zinc is dependent on the variety and processing method used. The information from this study can be used by food scientists and nutritionists in choosing the appropriate processing to increase the retention of high levels of micronutrient in yams and by the yam breeders to adjust their germplasm breeding activities.

## 1 Introduction

Iron and zinc deficiencies are common in the people of developing countries either because of an insufficient intake of the micronutrients or their poor absorption from food. Zinc plays a major role in biochemical functions, giving it a unique role in growth and development (Flynn, 1992). It protects the skin and improves resistance to infection, disease, inflammation, and allergies (Tolonen, 1990). Ramakrishnan (2002) reported that more than half of the world's population is at a risk of low zinc intake. Iron, on the other hand, is important for energy production, immune defense, and thyroid function among others (Roser, 1986). Many countries in sub‐Saharan Africa (SSA) have the largest prevalence and the largest absolute numbers of micronutrient deficient individuals, with women and children most severely affected (Black et al., 2008; World Health Organization, 2009). In addition, more than 40% of children younger than 5 years old are estimated to suffer from iron deficiency and anemia in developing countries part of the world (De Onis, Frongillo, & Blössner, 2000; World Health Organization, 2000).

Malnutrition can lead to several health consequences including impaired cognitive and physical development. It has a long‐term effect, which unfortunately may be irreversibly established early in life and may result in retarded linear growth or stunting, lower cognitive abilities, poor attention, and higher rates of infection. This further affects educational progress, decreases work capacity, and life expectancy, thereby affecting economic growth (Grantham‐McGregor & Ani, 2001). The compound effect has immense economic cost implications. Solutions to address micronutrient deficiencies have been by food fortification and supplementation approaches through intervention programs. Even though effective, these programs are not easily sustainable by the beneficiaries in the long‐term. Food‐based approaches to combat micronutrient malnutrition are more likely to be sustainable in the long‐term. In particular, in countries where plant‐based foods are major staples, using improved varieties with higher iron and zinc content could effectively enhance micronutrient intake because they are available, affordable, culturally acceptable, and preferred. This will be important in developing countries where meeting the Recommended Dietary Allowance (RDA) of micronutrient is far from being achieved (Welch & Graham, 1999).

Yam is a staple crop in areas where it is grown (Asiedu, Ng, Vuylsteke, Terauchi, & Hahn, 1992) with over 90% of global production coming from West Africa with Nigeria the leading producer (FAO, 2003). Yam is consumed in different ways, such as boiled, fried, or baked products. Tubers are often dried and milled into flour for reconstituting into a stiff paste, which is eaten with soup. It is an elite crop and preferred over other crops in regions where it is cultivated. It can be stored longer than other root and tuber crops, e.g., 6 months, ensuring a food supply even at times of general scarcity. Yam is of major importance in the diet and economic life of people in West Africa, the Caribbean islands, Asia, and Oceania (Girardin et al., 1998; Ravindran & Wanasundera, 1992). Information on the nutritive value of yam has been previously highlighted (Afoakwa & Sefa‐Dedeh, 2001; Alves, 2000; Bradbury & Holloway, 1988; Opara, 1999). However, information is limited on the micronutrient (iron and zinc) content of yam and how much is retained when it is processed. The present work looked at iron and zinc retention during yam processing.

## 2 Materials and Methods

Fifteen landrace varieties in the collection of the yam breeding program were investigated. Tubers were harvested at full shoot senescence from a replicated trial at the International Institute of Tropical Agriculture (IITA), Ibadan. All the varieties originated from West Africa. Three healthy tubers of different sizes (large, medium, and small) were randomly selected from a pile of harvested variety and replication resulting in six tubers sampled for each variety. The weight of the tubers ranged from 1.2 to 2.0 kg. On average, one to three tubers are harvested per stand.

To obtain a representative sample of each yam variety under investigation and pair the samples for the retention study, the randomly selected three healthy tubers were washed with tap water, peeled, rinsed with deionized water, and cut longitudinally (from the stem end to the tuber end) into four equal parts. Opposite quarters were taken for the raw sample and the other two opposite quarters for the processed sample, combining all the opposite quarters making up the sample for raw and for the processed products.

### 2.1 Food product preparation

#### 2.1.1 Boiled yam

The opposite quarters were chopped into chunks, combined, and mixed. The mixed sample was further divided into four equal parts. Adjacent sections were collected. A subsample of 200 g cut slices was cooked with 400 ml of water for 20 min. Cooked slices were drained, packaged in polythene whirl‐pack, and kept at −80°C until laboratory analysis for physicochemical composition, iron and zinc content. The cooked pieces were thawed at room temperature, and homogenized to a homogenous pulp mixture before aliquots were weighed out for analysis. The remaining quarters were used for the raw analysis resulting in a paired sample.

#### 2.1.2 Yam flour

A quantity of 500 g of the remaining yam chunks from the opposite quarters used for the boiled yam was immersed in 1 L of hot water (63 ± 3°C) and left covered in plastic containers for 24 hr. The slightly fermented slices were oven‐dried at 45°C for 72 hr and milled into flour, locally called *elubo* in Nigeria, packaged in polythene whirl‐pack, and kept at −80°C until laboratory analysis for physicochemical composition, iron and zinc content.

### 2.2 Laboratory analysis

#### 2.2.1 Preparation of flour for physicochemical analysis

The opposite quarters that were taken representing the raw sample were chopped into smallv pieces, combined, and mixed. The mixed sample was further divided into four equal parts. Adjacent sections were collected, mixed thoroughly, and dried in an air convection oven at 60°C for 72 hr. The dried pieces were milled into fine flour and packaged in polythene whirl‐pack, and kept at −20°C until laboratory analysis for physicochemical composition.

#### 2.2.2 Determination of physicochemical composition

Frozen samples were allowed to thaw to room temperature before duplicate subsamples were taken for analysis. The moisture and ash content of the fresh (raw) and food product samples were determined using standard AOAC (1990) methods as described.

Protein content was determined using the Hach (1990)method. To 0.20 g sample in a digestion tube, concentrated sulfuric acid (5 ml), hydrogen peroxide (10–15 ml), and one catalyst tablet were added. The mixture was digested at 375°C for 3 hr. Distilled water was added to the digest to a final volume of 75 ml, covered with paraffin, and mixed thoroughly. One ml of the mixture was pipetted into a 25 ml volumetric flask; three drops each of mineral stabilizer and polyvinyl alcohol solution were added and made up to volume with distilled water. Nessler reagent (1 ml) was added for color development. Absorbance was read at 460 nm using a HACH‐DR/3000 spectrophotometer (HACH Company, Loveland, CO) to determine the nitrogen concentration. Protein content was calculated using a factor of 6.25.

The starch and total sugar content were determined using a colorimetric method (Dubois, Gilles, Hamilton, Rebers, & Smith, 1956). Absorbance of the sample was read against a reagent blank at 490 nm using a Spectronic 601 spectrophotometer (Milton Roy Company, Ivyland, PA).

Amylose content was determined using the method described by Juliano (1971) involving the preparation of a stock iodine solution. Absorbance of the sample was read against a reagent blank at 620 nm using a Spectronic 601 spectrophotometer.

#### 2.2.3 Determination of iron and zinc retention

The homogenized samples of raw and boiled yam were thawed to room temperature, placed in Petri dishes, dried in an uncorroded oven at 40°C for 3 days and ground to flour with an approximate particle size of 0.5 mm. Oven‐dried aliquots of the raw, boiled and yam flour samples were sent to Waite Analytical Services, Adelaide, Australia for determination of the Fe and Zn content using Inductively Coupled Plasma Atomic Emission Spectrometry (ICPAES) (Choi, Graham, & Stangoulis, 2007). One 0.6 g sample of the products was cold digested in 50 ml tubes overnight using 11 ml of nitric/perchloric acid mixture (10:1) and made to a final volume of 25 ml with water. Duplicate aliquots of the digested samples were analyzed by ICPAES (model 3580 B; ARL, Switzerland) using a method described by Zarcinas, Cartwright, and Spouncer (1987). Values reported are the mean from duplicate aliquot determinations of each replication. A certified standard reference sample was randomly included to check for contamination (Choi et al., 2007).

The true retention (TR) of Fe and Zinc was calculated as follows (Murphy, Criner, & Gray, 1975):

$$\%\mathrm{TR}=\frac{\mathrm{Iron}\;\mathrm{or}\;\mathrm{zinc}\;\mathrm{per}\;g\;\mathrm{of}\;\mathrm{cooked}\;\mathrm{food}\;x\;g\;\mathrm{of}\;\mathrm{food}\;\mathrm{after}\;\mathrm{cooking}}{\mathrm{Iron}\;\mathrm{or}\;\mathrm{zinc}\;\mathrm{per}\;g\;\mathrm{of}\;\mathrm{raw}\;\mathrm{food}\;x\;g\;\mathrm{of}\;\mathrm{food}\;\mathrm{before}\;\mathrm{cooking}}x100$$

### 2.3 Statistical analysis

The general linear model procedure (GLM) of SAS version 8e, (SAS, 2001) was used for analysis of variance (ANOVA) at *p* < .05. Comparison among the treatment averages was by the least significance difference test (LSD) at the level of 5% of probability. Pearson correlation analyses were conducted to establish if there is a relationship between raw iron and zinc content and in the processed product (boiled yam and flour).

## 3 Results and Discussion

The physicochemical composition of the processed products, boiled yam, and yam flour of 15 *D. rotundata* varieties are presented in Tables 1 and 2. For boiled yam (Table 1), the overall mean values obtained on dry weight basis of edible portion were as follows: protein 5.6 g/100 g, amylose 24.1 g/100 g, total sugar 3.0 g/100 g, starch 74.6 g/100 g, and ash 2.4 g/100 g. The overall mean moisture content of the fresh yam tubers was 69.3%. The overall mean of the physicochemical composition for yam flour were: moisture 8.3 g/100 g, protein 5.4 g/100 g, amylose 23.7 g/100 g, total sugar 3.2 g/100 g, starch 62.4 g/100 g, and ash 2.6 g/100 g. Significant variations (*p* < .05) were observed among the varieties in many of the parameters determined. However, no wide variations were observed between the boiled and flour products in terms of the physicochemical composition with the exception of moisture and dry matter content, as expected.

**Table 1:** Physicochemical composition (g/100 g edible portion) of boiled yam of 15 *D*. *rotundata* varieties^a^

Variety	Moisture (g/100 g)	Dry matter (g/100 g)	Protein (g/100 g)	Amylose (g/100 g)	Total sugar (g/100 g)	Starch (g/100 g)	Ash (g/100 g)
TDr 97/00205	74.6^a^	25.5^g^	5.4^fg^	16.8^g^	4.0^bc^	66.1^g^	2.6^ed^
TDr 97/00960	70.4^cd^	29.7^de^	6.3^b^	16.7^g^	3.0^e^	73.5^de^	2.2^h^
TDr 97/00903	73.8^ab^	26.2^fg^	4.4^i^	18.0^f^	1.0^h^	77.3^bc^	1.9^i^
TDr kokumo	68.4^d^	31.7^d^	5.8^ced^	25.6^c^	3.0^e^	79.6^ab^	2.4^gh^
TDr 97/00777	68.5^d^	31.5^d^	7.5^a^	22.9^e^	1.4^hg^	63.2^h^	2.8^bc^
TDr 96/01724	73.4^ab^	26.6^fg^	4.9_h_	23.4^e^	1.5^g^	75.8^dc^	2.9^b^
TDr 96/01799	70.3^cd^	29.7^de^	6.1^cbd^	42.8^a^	2.5^f^	71.7^ef^	2.4^fg^
TDr omi‐efun	65.4^ef^	34.6^bc^	6.2^bc^	24.5^d^	2.1^f^	76.1^cd^	2.5^ef^
TDr 97/01217	75.2^a^	24.8 ^g^	4.7^hi^	27.5^b^	5.1^a^	78.3^abc^	1.7^j^
TDr 96/01395	68.6^d^	31.4^d^	5.5^ef^	25.3^c^	2.3^f^	69.6^f^	3.1^a^
TDr 96/01393	67.8^de^	32.2^cd^	5.7^edf^	18.3^f^	3.8^cd^	75.9^dc^	2.8^bc^
TDr 96/00428	65.6^ef^	34.4^cb^	5.6^ef^	23.4^e^	3.5^d^	76.3^c^	2.7^cd^
TDr 97/00588	64.3^fg^	35.7^ab^	6.1^cbd^	24.9^cd^	3.0^e^	75.7^dc^	2.3^gh^
TDr 96/01750	61.7^g^	38.3^a^	5.5^ef^	25.2^cd^	4.7^a^	79.7^ab^	1.8^j^
TDr 96/01817	71.4^bc^	28.6^ef^	5.0^gh^	25.6^c^	4.3^b^	80.8^a^	2.6^ed^
Mean	69.3	30.7	5.6	24.1	3.0	74.6	2.4
SE	1.15	1.15	0.22	1.81	0.36	1.46	0.12
LSD	2.66	2.66	0.38	0.79	0.45	2.61	0.13

a Values are mean of duplicate analyses of a composite sample of three yam tubers from a replicated trial of the same harvest batch boiled for 20 min. Means with same superscripts in the same column are not significantly different at 5% level of significance. LSD, least significance difference.

**Table 2:** Physicochemical composition (g/100 g edible portion) of yam flour of 15 *D. rotundata* varieties^a^

Variety	Moisture (g/100 g)	Dry matter (g/100 g)	Protein (g/100 g)	Amylose (g/100 g)	Total sugar (g/100 g)	Starch (g/100 g)	Ash (g/100 g)
TDr 97/00205	9.3^b^	90.7^g^	5.0^de^	22.7^f^	4.1^bc^	69.2^b^	2.6^d^
TDr 97/00960	8.9^c^	91.2^f^	5.5^cd^	24.9^d^	3.2^cde^	54.9^ef^	3.0^b^
TDr 97/00903	9.8^a^	90.2_ _^h^	5.0^de^	24.5^ed^	2.3^efg^	58.7^cde^	3.0^b^
TDr kokumo	8.6^cde^	91.4^edf^	6.4^a^	23.0^f^	1.9^g^	60.9^cde^	2.5^d^
TDr 97/00777	8.9^c^	91.1^f^	6.3^a^	19.5^h^	3.1^de^	64.9^bc^	3.0^b^
TDr 96/01724	8.4^de^	91.6^cd^	5.3^cd^	20.0^h^	2.5^efg^	64.1^bc^	2.7^c^
TDr 96/01799	8.7^cd^	91.3^ef^	5.3^cd^	19.4^h^	3.2^cde^	76.7^a^	2.3^e^
TDr omi‐efun	7.0^g^	93.0^b^	4.6^e^	24.4^ed^	5.3^a^	62.7^c^	1.4^g^
TDr 97/01217	7.5^f^	92.5^c^	5.3^cd^	29.2^a^	1.6^g^	50.7^f^	2.2^e^
TDr 96/01395	6.5^h^	93.5^a^	6.3^a^	23.9^e^	2.1^fg^	55.1^ef^	2.5^d^
TDr 96/01393	7.5^f^	92.5^c^	6.2^a^	20.9^g^	2.9^def^	78.4^a^	3.4^a^
TDr 96/00428	8.2^e^	91.8^d^	6.1^ab^	24.1^e^	3.2^cde^	55.9^def^	3.0^b^
TDr 97/00588	8.7^cd^	91.3^ef^	3.9^f^	28.2^b^	5.0^ab^	62.0^cd^	2.0^f^
TDr 96/01750	8.6^cd^	91.4^ef^	4.6^e^	26.1^c^	4.8^ab^	60.5^cde^	3.0^b^
TDr 96/01817	8.7^cd^	91.3^ef^	5.6^bc^	24.1^e^	3.6^cd^	60.9^cde^	2.5^d^
Mean	8.3	91.7	5.4	23.7	3.2	62.4	2.6
SE	0.25	0.25	0.22	0..84	0.33	2.22	0.14
LSD	0.42	0.42	0.54	0.61	0.98	6.47	6.12

a Values are mean of duplicate analyses of flour from a composite sample of three yam tubers from a replicated trial and of the same harvest batch. Means with same superscripts in the same column are not significantly different at 5% level of significance. LSD, least significance difference.

The yam tuber, in general, has high moisture and low dry matter content. Limited knowledge about storage and processing technologies available to the communities consuming yam result in losses of the crop during the harvesting season. However, yam is usually processed into flour consumers for use in various food products such as *amala*, a thick paste made from yam flour.

The very low moisture content of yam flour observed in this study shows the value of the crop in terms of food security because it can be stored and used throughout the year. Appropriately stored yam flour could therefore be “stored wealth” for the farmers and processors who are involved with yam production and trade in the regions where it is grown as a food crop.

Similar observations in protein content (5.4–8.0 g/100 g as is) and dry matter (40.4–26.4 g/100 g) of yam have been reported (Treche & Agbor‐Egbe, 1996). Protein content is, however, higher than that reported in cocoyam and cassava (Sefah‐Dedeh and Agyir‐Sackey, 2002).

Yam contains no cyanogenic compounds; it has higher protein and vitamin C content compared to cassava which makes it an important crop from a nutritional point of view among the root and tuber crops. Comparable ranges from 0.8 to 16.4 g/100 g of sugar and 59.9% to 78.9% of starch have been reported in high yield and disease‐resistant varieties of yam (Maziya‐Dixon & Asiedu, 2003). Differences in sugar and total starch content in the varieties analyzed in this work could be due to differences in the activity of enzymes involved in starch biosynthesis (Krossmann & Lloyd, 2000). Starch accounts for more than 80 g/100 g on a dry weight basis of yam carbohydrate; hence, it is a dominant factor in determining the physico‐chemical, rheological, and textural characteristics of yam food products. Starch owes much of its functionality to the amylose/amylopectin ratio, which may influence the pasting and physicochemical properties such as viscosity, retrogradation, solubility, or water absorption. As a result, the food and industrial uses of starches is very much dependent on this ratio (Moorthy, 1994; Scott, 1996). Similar amylose content ranging from 14 to 30 g/100 g to what was observed in this study has been reported for *D. rotundata* (Bokanga, 2000; Moorthy, 2002). Information on the chemical composition of yam therefore, is essential in accessing the suitability of yam tubers for processing into flour for different products.

Tables 3 and 4 present the retention of iron and zinc in boiled yam and yam flour, respectively. The retention of iron in boiled yam varied from 56% to 99% (9.1–14.6 mg/kg) and from 25% to 55% (11.2–20.5 mg/kg) in yam flour. The retention of zinc in boiled yam and yam flour were 49–98% (6.7–13.4 mg/kg) and 19–43% (5.7–14.8 mg/kg), respectively. The TDr 97/00903 variety retained the least amount of iron in both the boiled yam and yam flour while the TDr 97/00588 (boiled yam) and TDr 96/01750 (yam flour) varieties retained the most amount of iron. In boiled yam, TDr 96/01393 had the highest zinc retention and in yam flour, TDr 97/00205 retained the most zinc.

**Table 3:** Retention of iron and zinc in boiled yam of 15 *D*. *rotundata* varieties^a^

Variety	Fe content (mg/kg yam)		% TR^b^	Zn content (mg/kg yam)		% TR^b^
	Raw	Boiled	Fe	Raw	Boiled	Zn
TDr 97/00205	12.0	12.1	98.3	14.0	13.4	93.2
TDr 97/00960	21.0	14.6	70.8	14.0	13.4	97.4
TDr 97/00903	17.0	13.5	55.5	14.0	11.4	57.1
TDr kokumo	15.0	10.1	65.1	12.0	11.5	92.9
TDr 97/00777	12.0	12.4	69.0	15.0	11.1	49.3
TDr 96/01724	13.0	12.2	97.0	9.8	9.2	96.5
TDr 96/01799	10.0	9.9	74.0	10.0	10.1	75.6
TDr omi‐efun	11.0	10.4	96.9	7.5	6.7	91.2
TDr 97/01217	12.0	11.4	92.2	8.8	8.4	91.8
TDr 96/01395	10.0	9.1	93.5	12.0	11.0	94.8
TDr 96/01393	16.0	12.0	94.1	12.0	11.3	97.5
TDr 96/00428	14.0	12.4	88.9	9.8	8.6	88.0
TDr 97/00588	11.0	10.9	98.7	14.0	12.0	78.4
TDr 96/01750	9.8	10.9	96.5	8.3	8.2	85.3
TDr 96/01817	9.9	10.0	95.9	11.0	11.0	95.2
Min	9.8	9.1	55.5	7.5	6.7	49.3
Max	21.0	14.6	98.7	15.0	13.4	97.5
Mean	12.9	11.5	85.8	11.5	10.5	85.6
SE	0.8	0.4	3.8	0.6	0.5	3.8

a Values are mean of duplicate aliquot determinations per variety and replication.

b Percent true retention. TR, true retention.

**Table 4:** Retention of iron and zinc in yam flour of 15 *D*. *rotundata* varieties^a^

Variety	Fe content (mg/kg yam)		% TR^b^	Zn content (mg/kg yam)		% TR
	Raw	Flour	Fe	Raw	Flour	Zn
TDr 97/00205	12.0	20.5	45.8	14.0	14.8	43.1
TDr 97/00960	21.0	20.3	38.6	14.0	12.7	36.3
TDr 97/00903	17.0	16.1	25.2	14.0	14.1	26.8
TDr kokumo	15.0	14.3	28.7	12.0	9.5	23.7
TDr 97/00777	12.0	15.5	44.7	15.0	11.3	26.1
TDr 96/01724	13.0	12.3	28.5	9.8	6.9	21.2
TDr 96/01799	10.0	16.0	44.3	10.0	14.0	42.0
TDr omi‐efun	11.0	12.1	44.0	7.5	5.7	18.9
TDr 97/01217	12.0	13.1	39.7	8.8	7.6	21.1
TDr 96/01395	10.0	11.2	32.8	12.0	9.4	33.9
TDr 96/01393	16.0	13.6	47.7	12.0	8.6	25.2
TDr 96/00428	14.0	14.4	48.1	9.8	7.9	26.4
TDr 97/00588	11.0	11.8	40.3	14.0	11.6	31.0
TDr 96/01750	9.8	12.6	54.9	8.3	7.3	37.3
TDr 96/01817	9.9	11.7	44.5	11.0	9.7	33.0
Min	9.8	11.2	25.2	7.5	5.7	18.9
Max	21.0	20.5	54.9	15.0	14.8	43.1
Mean	12.9	14.4	40.5	11.5	10.1	29.7
SE	0.8	0.7	2.2	0.6	0.7	2.0

a Values are mean of duplicate aliquot determinations per variety and replication.

b Percent true retention. TR, true retention.

Retention of both iron and zinc was comparably higher in boiled yam than in yam flour samples. The lower retention in yam flower could be as a result of the 24 hr period during which the tuber slices/pieces were kept in hot water for parboiling and slight fermentation, resulting in the leaching of nutrients (minerals or mineral compounds) into the water. Losses in minerals or mineral compounds could also have occurred during the milling process as a result of an increased surface area of the more numerous amounts of particles resulting in the adherence to the milling instrument as opposed to the larger intact yam slices of the boiled samples. Boiling was reported to result in a reduction in iron and zinc content when groundnut (*Arachis hypogaea*) seeds were processed to a paste (Onyeike & Oguike, 2003). Thus, nutrient losses may depend on the edible surface area exposed during food processing.

In the traditional processing, yam tubers are peeled, cut into medium sized pieces, and cooked in water. This method of processing leads to leaching of minerals and other nutrients into the water, which is eventually discarded. The cooking of intact yam tubers without peeling may be recommended in order to retain more nutrients, especially the water‐soluble nutrients. Although cooking reduces the mineral content, it is also known that cooking increase nutrient bioavailability by decreasing antinutritional factors, such as phytate, known to inhibit mineral/micronutrient absorption. Marfo and Oke (1988) reported that oven‐drying has only a small reductive effect on the phytate content, unlike cooking which reduced the phytate content to 62% of the original content in yam.

A large variation was observed in the content of iron and zinc among the yam varieties reported in the present work. This is very promising indeed, suggesting prospects for the enrichment of yam varieties with minerals such as iron and zinc through breeding programs. There was a positive relationship between the amount of iron and zinc retained in yam flour (*R* = .72). A similar trend but a weaker relationship was observed between iron and zinc in boiled yam. This implies that varieties with higher iron content may likewise have higher zinc content.

The amount of iron and zinc retained in boiled yam positively correlated with the amount in raw samples, *r* = .80 and *r* = .90, respectively. A similar positive and strong correlation was observed in yam flour, *r* = .82 and *r* = .69 for iron and zinc, respectively. This suggests that yam varieties containing higher amounts of iron and zinc are likely to retain more in their products. The low retention of iron and zinc in yam flour demonstrates the importance of having mineral‐rich raw material in processed products. The results of the present work offer scientific information for breeding programs that aim to increase micronutrient content of iron and zinc in particular, in available, affordable, and preferred staple crops such as yam.

## 4 Conclusion

Boiling and flour preparation processes of yam varieties reduced the iron and zinc content to various degrees compared to the amount present in the fresh tubers. Processing yam tubers into flour had a greater reductive effect than boiling which retained as high as 85% iron and zinc. Iron and zinc retention varied across varieties and the amount retained in the products are related to the content in the fresh sample. The findings of this study showed that with the appropriate processing of yams, it could contribute significantly to the micronutrients intakes of low‐income communities of developing countries that widely consumed yams on a daily basis. The information obtained from this study will not only increase the understanding of the level of retention of Iron and zinc after processing but will also help breeders to adjust their germplasm development activities for high content of such micronutrient. This information can also help researchers in choosing proper processing methods to increase the retention of high levels of micronutrient in yams that can be delivered to consumers through nutrition education.

## Conflict of Interest

None declared.
